# Imaging Modalities for the Noninvasive Assessment of Fibrosis in Crohn's Disease

**DOI:** 10.1100/2012/450151

**Published:** 2012-04-30

**Authors:** Cristina Stasi, Massimo Falchini, Stefano Milani

**Affiliations:** ^1^Department of Internal Medicine, University of Florence, 50134 Florence, Italy; ^2^Department of Clinical Pathophysiology, University of Florence, 50139 Florence, Italy

## Abstract

The development of strictures in Crohn's disease is a main cause of hospitalization and often represent an indication for surgery. The differentiation between inflammatory and fibrotic strictures is useful to determine the optimal treatment. Today, the availability of noninvasive methods to assess the presence and extension of strictures offers new tools for the diagnosis and follow-up of the disease. Bowel ultrasound, power doppler ultrasound, contrast-enhanced ultrasound, magnetic resonance imaging offer the additional advantage that they do not expose patients to ionizing radiation. In this paper we provide an update on the accuracy of these noninvasive methods for the diagnosis of Crohn's disease.

## 1. Introduction

The incidence of Crohn's disease (CD) is increasing worldwide. The highest incidences have been reported in northern Europe, the United Kingdom, and North America. In the past 50 years, the incidence and prevalence in the West has increased up to 6–15/100,000 and 50–200/100,000 persons, respectively [1].

CD is a chronic inflammatory condition of the gastrointestinal tract, in which genetic, psychological, immune, and inflammatory factors have been suggested to play a role in its pathogenesis [2].

The physiological immune response in the gut to suppress a possible over reactivity bowel antigens or antigens introduced by food. Pizarro et al. [3] suggest that local TNF production by intestinal epitelial cell may be key in maintaining proper innate immune defences. Early deterioration of its expression can lead to increased bacterial penetration and exposure of underlying mucosal immune cells to commensal antigens, thus triggering the onset of intestinal inflammation.

The transmural fibrogenic process observed in CD is believed to be induced by a complex interplay between inflammatory and fibrogenic cells. As for other diseases characterized by fibrosis, myofibroblasts have been suggested to play a key role in intestinal fibrosis. However, most of the assumptions on the pathophysiology of fibrosis in CD are based on data analyzing the fibrogenic process occurring in other organs.

Strictures are often responsible of patient's hospitalizations and surgeries [4]. Histologically, strictures contain a mixture of inflammatory and mesenchymal cells with deposition of an excess of extracellular matrix, resulting in different degrees of fibrosis [5]. In fact, strictures may be predominantly inflammatory or fibrotic, causing diagnostic and therapeutic uncertainty. Treatment regimens include immune modulators, but this therapy fails when there is intestinal fibrosis [6]. This would require clinical assessment with noninvasive methods to determine the activity and extent of disease and to assess intestinal fibrosis.

Bowel ultrasound is useful to detect intestinal and extraintestinal lesions, including bowel wall thickening, stenosis with or without bowel dilation, and the presence of abscesses and fistulae. Contrast-enhanced ultrasound (CEUS) may show an enhancement of the bowel wall signal associated with disease activity. In particular, in patients with stenotic segments and incomplete bowel obstruction, it is very important to distinguish between fibrosis and inflammation (enhancement with CEUS). These diagnostic techniques do not expose patients to ionizing radiation hazards and may be repeated frequently during the monitoring of therapy, particularly to exclude the need for surgery.

Magnetic resonance imaging (MRI) has also been proposed as a useful tool to distinguish between intestinal inflammation and fibrosis. Similarly to bowel ultrasound, it does not expose to ionizing radiation, but its costs and accessibility might preclude its frequent use in the monitoring of patients. On the other hand, one of the main advantages of MRI is that it provides more detailed information about disease involvement of the small bowel wall and possible extraintestinal complications [7].

This paper provides an update of non-invasive diagnosis of Crohn's disease, focusing on the role of transabdominal ultrasound and magnetic resonance in the detection of intestinal fibrosis in Crohn's disease. The aim is to discuss the accuracy of abdominal ultrasound and magnetic resonance in Crohn's disease in order to establish a proper diagnostic assessment and optimize treatment.

## 2. Assessment by Transabdominal Ultrasound

Improvements in technology and increasing experience with ultrasound in inflammatory bowel disease (IBD) have confirmed the role of ultrasound as a clinical tool in Gastroenterology Units. Transabdominal ultrasound is clinically useful as a non-invasive imaging modality in the diagnosis of IBD by evaluating bowel wall thickness, peri-intestinal inflammatory reaction, and by estimating extraluminal complications such as stenosis, fistula, and abscesses. For these reasons, transabdominal ultrasound has been proposed as a first-line diagnostic tool in assessing patients with Crohn's disease regardless of their clinical symptoms and/or disease activity [7, 8]. In recent studies, sensitivity of transabdominal ultrasound in detecting Crohn's disease ranges from 84 to 93% when compared with endoscopy and/or radiology as gold standard [9–11]. Specificity is 98%, in the study of Astegiano and coworkers [9] performed on 313 consecutive outpatients presenting with abdominal pain and irregular bowel habits lasting more than 3 months. It has been suggested that disease location is the main factor influencing the accuracy of ultrasound for the diagnosis of CD. Sensitivity is lower for less accessible locations such as rectum (14.2%) and upper small bowel (28.6%) [10].

**Figure 1:**
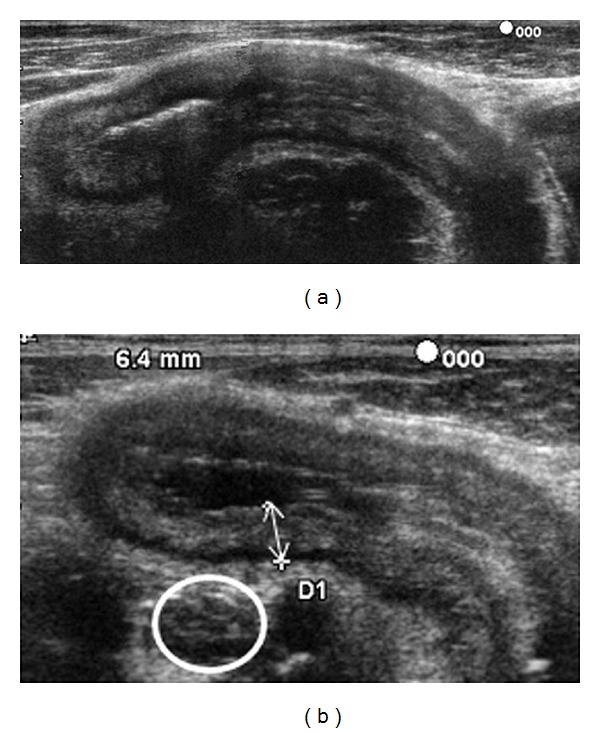
(a) Thickening of the ileum wall (6.4 mm). Maintained laminar aspect of the intestinal wall and (b) enlarged lymph node (white circle) near the terminal ileum.

The initial diagnosis of CD may be supported by ultrasound measurement of bowel wall thickness. Most studies reported normal wall thickness ranging from 1 up to 4 mm [12]. As shown in Figure 1, the ileum wall echotexture (high-resolution transducers usually permit visualization of five layers) and the possible involvement of contiguous structures could also be informative and should be considered [8].

In particular, a correlation has been found between wall thickness and disease activity. Wall thickness is significantly higher in active (Crohns Disease Activity Index, CDAI > 150) than in inactive disease (CDAI < 150). Moreover an association between fibrosis and wall thickening was found. This association is important for differential diagnosis of inflammatory and fibrotic bowel wall changes. Fibrotic stenoses are mostly represented in segments with wall thickening <20–30 mm, whereas inflammatory lesions are more frequent in segments with >30 mm wall thickening [8]. The addition of an oral nonadsorbable solution (polyethylene glycol) results in an increase in the sensitivity of ultrasound in defining anatomic location and extension of segments with active disease [13, 14].

## 3. Assessment by Power Doppler and Contrast-Enhanced Ultrasound

Newer techniques such as power Doppler and the administration of echo-enhancing agents have further improved sensitivity and accuracy.

A strong correlation between colour Doppler sonographic vascularity and colonoscopy and histology of the terminal ileum and the right colon was found [15, 16].

These studies suggest the utility of power Doppler ultrasound in the assessment of activity changes of the bowel wall determined by pharmaceutical treatment.

In patients with CD, the analysis of vascularity after intravenous injection of Levovist increases the accuracy of transabdominal ultrasound and may facilitate the differentiation between inflammatory and fibrotic stenosis [17, 18].

Other studies [19, 20] employing second-generation intravenous contrast agent (SonoVue) reported similar results in the accuracy of CEUS.

Different diffusion-perfusion patterns of bowel wall enhancement can be observed in CD [19, 20], reflecting different degrees of disease activity: submucosal prevalent enhancement, transparietal enhancement starting from the submucosa, and transparietal enhancement pattern starting from extravisceral vessels and involving the whole wall with an external-to-internal direction. These patterns may represent the basis for a semiquantitative method to assess wall vascularisation. Serra et al. [19] described a quantitative method based on the E/W ratio, in which E is the major thickness of the enhanced layer, and W is the thickness of the entire wall section.

CEUS is a sensitive tool for the determination of disease activity and it was seen to be useful in the followup of treatment. In addition, this technique has been helpful in surgical management, particularly in the decision of conservative versus surgical treatment [21, 22]. In fact, hypervascularity of stenosed segments was shown in intestinal obstruction subsequent to inflammatory cause, while hypovascularity was found in cicatricial stenosis [23].

In a recent study [24] intestinal fibrosis was evaluated by measuring strain (degree of compression of a material in response to a force applied to a fixed area), that is developed in the tissue. In the study, 7 consecutive patients with stenosed segment were studied with ultrasound elasticity imaging. Their stenotic and normal bowel segments were evaluated by ex vivo elastometry and histopathology after surgical resection. In addition, female Lewis rats undergoing weekly trinitrobenzene (5 with subsequent acute inflammatory colitis and 6 with chronic intestinal fibrosis) were evaluated. The study showed that ultrasound elasticity imaging can differentiate inflammatory from intestinal fibrosis in rat models, as well as fibrotic lesions from unaffected intestine in patients with CD.

Ultrasound elasticity imaging could identify the patients with high risk to develop fibrosis and rapidly progress to fibrostenotic strictures. Therefore, it could be useful in treatment decision, but future prospective clinical studies are needed.

## 4. Assessment by Magnetic Resonance Imaging

MRI provides information about complications and extraintestinal manifestations, without exposure to ionizing radiation. The major advantage of MRI over US is the assessment of the entire gastrointestinal tract [25]. On the contrary, the disadvantages are the accessibility to this imaging modalities, greater cost, longer interpretation time and duration of the exam, contraindication such as claustrophobia, pacemaker, ferromagnetic vascular clips, ferromagnetic foreign body in the noble (intracranial, intraocular or vascular), Swan-Ganz catheter.

MRI is able to provide a rapid and accurate evaluation of the abdomen and pelvis. In particular, it can be helpful in providing an evaluation of the activity of perianal CD [26]. It assesses the features of an altered bowel segment through characteristic patterns of attenuation and degrees of enhancement.

Some technical aspects, in particular the distension of the bowel, enhance the accuracy of MRI. For this reason a large amount of oral contrast is used in MRI examination. Oral contrast also decreases the susceptibility to artifacts by displacing intraluminal air. Positive contrast agents, represented by paramagnetic substances such as gadolinium chelates, increase intraluminal signal (hyperintense on both T1-weighted and T2-weighted). On the contrary, negative contrast agents reduce intraluminal signal. Moreover, they improve the visualization of inflamed bowel wall and surrounding fat on T2 images [27]. Bowel wall thickening is detected on T2 images and bowel wall enhancement is better appreciated on T1 images. Fat saturation may be used to increase contrast resolution. This technical aspect also allows a better assessment of bowel enhancement [28, 29].

Bowel wall thickness can be scored as normal (< 3 mm), mildly abnormal (3–6 mm), or markedly abnormal (> 6 mm) [30]. The changes characteristically associated with disease activity are the increase of wall thickness and the enhancement after injection of MRI contrast and presence of oedema.

**Figure 2:**
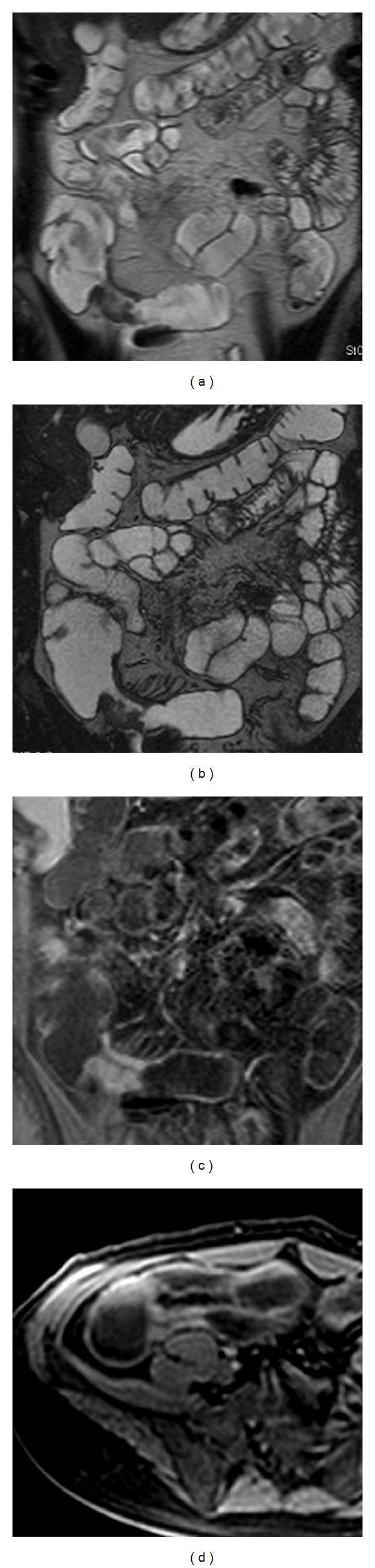
(a) MR enterography, T2 weighted sequence (HASTE, half-Fourier axial single-shot fast spin-echo): relapse of segmental disease associated with segmental wall thickening at the site of previous ileocolic anastomosis; (b) MR enterography T2 weighted sequence (TrueFISP, true fast imaging with steady-state free precession) delineated profiles of segmental wall thickening and mild *comb* sign; (c-d) MR enterography, T1-weighted sequence (VIBE, volumetric interpolated breath-hold examination): postcontrast homogeneous enhancement of the intestinal wall suggesting a fibrotic lesion.

Borthne et al. [11] compared ultrasound and MRI using ileocolonoscopy as reference standard in 53 paediatric patients with suspected IBD. Ultrasound showed higher sensitivity than MRI (93% versus 82%), but only the terminal ileum, the location with the highest accuracy for ultrasound, was examined. In a tertiary care medical center, 120 patients with diagnosis of CD or subjects who were suspected of having CD underwent MR enterography. MR enterography demonstrated active CD in 57% of patients, chronic changes of CD without active inflammation (e.g., stricture, fistula, or abscess) in 12.5% of cases. The authors demonstrated that MR Enterography can add significant information to the clinical evaluation of these patients and were useful to determine if new surgical or medical treatment was required [31].  Figure 2 shows the relapse of disease after resection and primary anastomosis.

In another tertiary medical center, a study on 213 patients during 4-year showed that 60% of MR Enterography presented bowel wall thickening and 53% displayed a clear signal enhancement [32].

Several studies [33–37] have evaluated the accuracy of MRI in the assessment of activity in the terminal ileum and/or the colon, showing that a higher accuracy for the assessment of disease activity can be obtained using luminal negative contrast.

Del Vescovo et al. [33] studied 16 CD patients who underwent ileocolonoscopy with biopsy and MRI. The authors observed a strong correlation between MRI and histological findings. Bowel segments with active disease presented a layered pattern of enhancement, while inactive disease presented homogeneous contrast uptake. In active disease, during intravascular phase of contrast distribution, the authors showed the predominance of vasodilatation over neoangiogenesis with a fast rise of the mucosa-submucosa (internal layers) enhancement curve. On the contrary, muscularis-serosa (external layers) enhancement curve showed a “slower and lower” trend. In inactive disease, the predominance of neoangiogenesis over vasodilatation, probably due to parietal repair of the chronic damage, resulted in similar trends of the mucosa-submucosa and muscularis-serosa curves in all phases. The absence of the enhancement curves between the two layers mucosa-submucosa and muscularis-serosa in intravascular distribution of contrast agent was likely due to the absence of vasodilatation.

**Figure 3:**
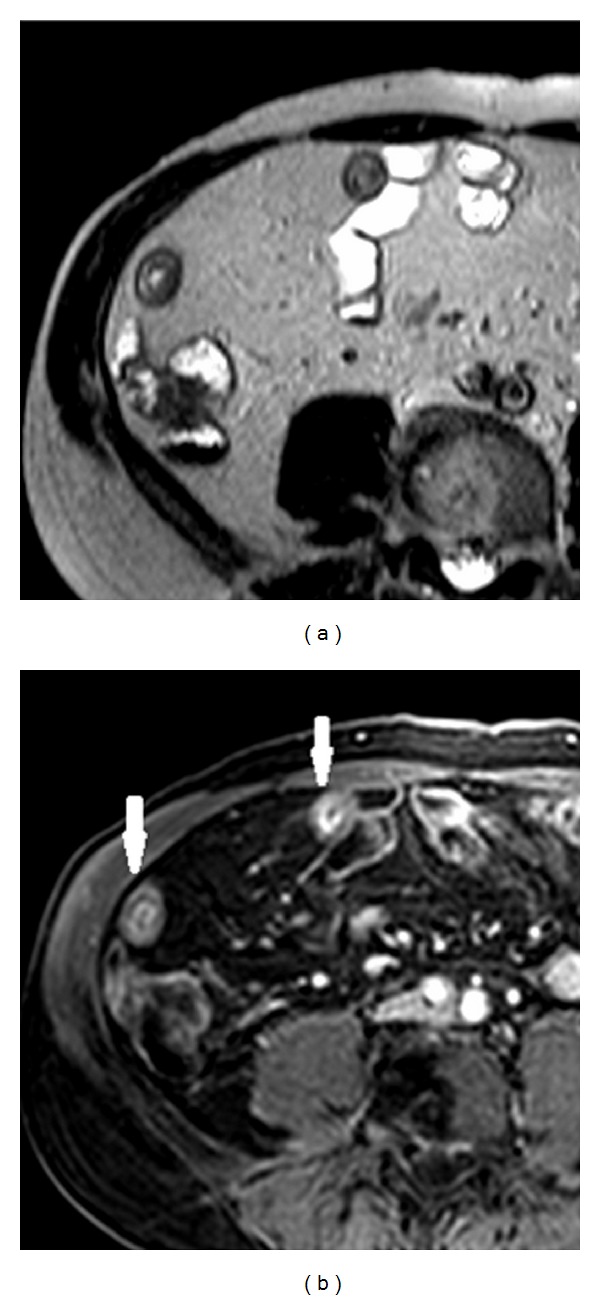
Active inflammation in Crohn's disease: (a) T2W sequence (HASTE): homogeneous wall thickening with intramural focal hyperintense signal area compliant with active inflammation; (b) postcontrast T1W sequence (VIBE) showing mucosal enhancement with laminar appearance of the wall (with arrows).

In Figure 3 the active inflammation in CD manifests as high signal intensity into the bowel wall.

Several studies confirm the possibility to distinguish the active inflammation from the chronic changes of fibrosis based on different enhancement patterns [5, 34, 35].

**Figure 4:**
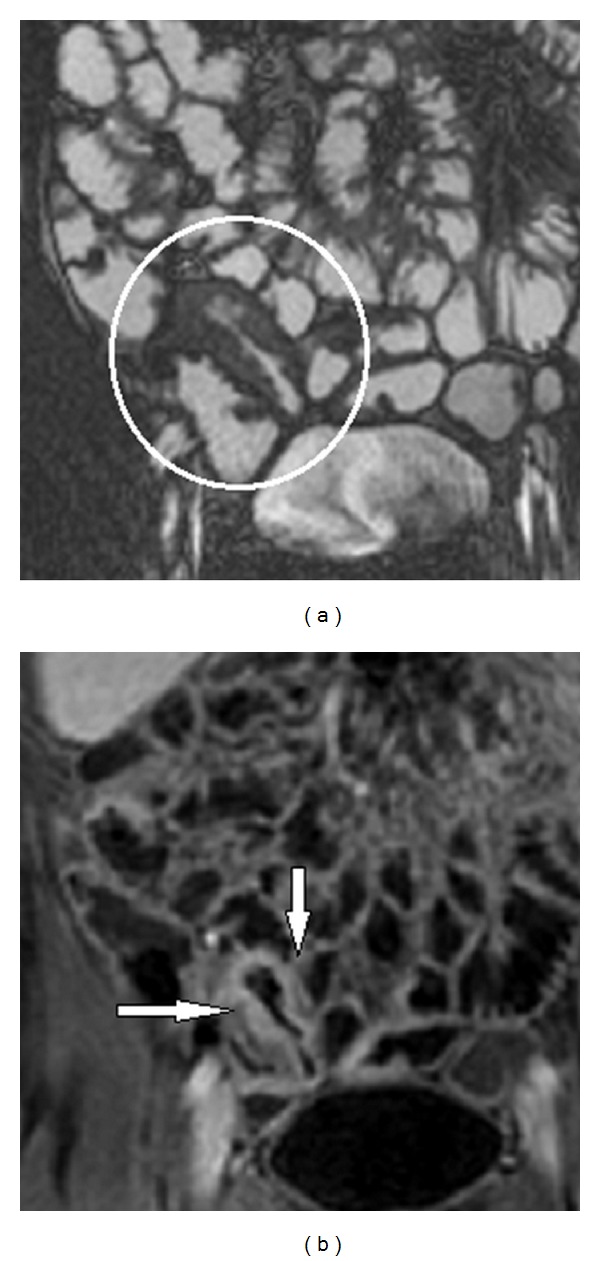
(a) HASTE sequence: mild inflammatory activity; distal ileum involved by disease with inhomogeneous wall thickening (white circle) (b) T1-weighted sequence (VIBE): postcontrast homogenous wall enhancement compliant with fibrotic evolution and reduced inflammatory activity (white arrows).

Figure 4 shows a chronic fibrotic stricture, in which there is homogeneous contrast enhancement without evidence of oedema or hyperaemia.

The study of Lasocki and colleagues [34], performed retrospectively in 26 patients with recent histology (surgery and/or colonoscopy), showed that MRI is most sensitive to detect active CD. Moreover, the most sensitive signs were those related to the bowel wall, namely, thickening, nodularity, contrast enhancement, and oedema. In the study of Ha et al. [35] 119 patients with obstructive symptoms were enrolled. Fifty-five % of patients required escalation of medical therapy and 32.5% were directed to surgery. All MR Enterography studies were performed in fasted patients followed by oral ingestion of a sorbitol-based biphasic contrast and it was given immediately prior to scanning the noncontrast sequences. A single dose of gadolinium contrast agent was administered before the postcontrast sequences. The presence of early postcontrast bowel wall enhancement and thickening in the setting of intermediate to increased T2 signal intensity bowel wall thickening was classified as active inflammation. The presence of fibrosis was suggested by delayed or absent contrast.

In the study of Lawrence et al. [36] response to continued medical therapy after MRI was prospectively assessed at 8-wk. Nonresponders underwent endoscopy. In patients undergoing surgical resection, the correlation between surgical pathology and the MRI was assessed, confirming the accuracy of MRI in the classification of both inflammation severity and the presence of fibrosis.

A recent study [5] was conducted to determine the utility of magnetization transfer (MT) MRI in the identification and quantification of intestinal fibrosis in a rat model of CD. Magnetization transfer MRI shows different molecular properties with respect to MRI. MT generates contrast that is primarily determined by the fraction of large macromolecules or immobilized phospholipids cell membranes in tissue. MT is able to determine the fibrotic content of small bowel strictures. Fibrosis progression was inducted in Lewis rats injected subserosally with peptidoglycan-polysaccharide. They develop bowel inflammation 1 day after laparotomy and fibrosis starting 14 days after laparotomy. The authors performed MRI in 25 rats injected with peptidoglycan-polysaccharide and 13 injected with human serum albumin (control animals). They showed that magnetization transfer can help detect fibrosis and that magnetization transfer ratio correlates with tissue collagen and it is sensitive to monitor changes in fibrosis progression into the natural history of CD.

## 5. Conclusions

Bowel ultrasound should be the first-line diagnostic modality for disease assessment and follow-up examinations with high accuracy. Ultrasound and MRI can be used in clinical practice as imaging modalities for assessment of fibrosis in the terminal ileum and colon. CEUS may be particularly useful in the followup of patients with CD and for the assessment of the efficacy of medical therapy in reducing bowel wall vascularity.

## Abbreviations

CD: Crohn's disease
CEUS: Contrast-enhanced ultrasound
MRI: Magnetic resonance imaging
IBD: Inflammatory bowel disease
CDAI: Crohn's disease activity index
MT: Magnetization transfer.
